# Refining and validation of Family Socioeconomic Status Scale (FSESS) for health research in Egypt

**DOI:** 10.1186/s12889-026-26282-y

**Published:** 2026-02-09

**Authors:** Abdel-Hady El-Gilany, Mohamed Baklola, Mohamed Terra, Merna Muhammad Harb, Sohila S. Gaballah, Elham S. Mohamed, Alwathiqbillah Abdelmeneam, Mohamed Abdelazez Hamada, Hala Samir Abou-ElWafa

**Affiliations:** 1 Public health and preventive medicine, faculty of medicine, Mansoura University, Mansoura, Egypt; 2 Faculty of medicine, Mansoura University, Mansoura, Egypt; 3 General practitioner, Ministry of health and population, Cairo, Egypt; 4 Faculty of veterinary medicine, South Valley University, Qena, Egypt; 5 Faculty of medicine, New Valley University, Al Khārijah, New Valley, Egypt; 6 Occupational Health and Industrial Medicine, Faculty of Medicine, Mansoura University, Mansoura, Egypt

**Keywords:** Socioeconomic status, Family socioeconomic scale, Egypt, Validation, Health research

## Abstract

**Background:**

Socioeconomic status (SES) is a key construct in public health and social research, yet its measurement varies across settings and over time. In Egypt, several existing SES scales are outdated and rely heavily on income, which has become increasingly unstable under current economic conditions. This study aimed to refine and psychometrically validate a concise Family Socioeconomic Status Scale (FSESS) that reflects contemporary socioeconomic conditions in Egypt.

**Methods:**

A community-based cross-sectional study was conducted using a multistage stratified cluster sampling design. Four governorates were selected to represent Egypt’s main geographic regions: Cairo (Greater Cairo), Dakahlia (Lower Egypt), Qena (Upper Egypt), and New Valley (Frontier Governorates). A total of 2,508 families were approached, 2,400 completed the survey (response rate 95.8%), and 2,090 were included in the final analysis. Data were collected through interviewer-administered Arabic questionnaires recorded electronically in English. The preliminary 20-item FSESS was developed based on literature review and expert consensus. Content validity was assessed by 13 public health experts. Exploratory and confirmatory factor analyses were performed using SPSS and R to assess construct validity and reliability.

**Results:**

Exploratory factor analysis demonstrated adequate sampling adequacy (KMO = 0.89; Bartlett’s test *p* < 0.001) and supported a three-factor structure comprising education, occupation, and family income and possessions, represented by six core indicators. These factors accounted for 49% of the total variance. Confirmatory factor analysis supported the three-factor model, with acceptable to good model fit across multiple indices (CFI and IFI > 0.90; RMSEA < 0.08). The overall internal consistency of the scale was good (Cronbach’s alpha = 0.82).

**Conclusion:**

The refined FSESS provides a concise and methodologically sound measure of family socioeconomic position within the studied Egyptian settings. By capturing multiple dimensions of SES while remaining feasible for large surveys, the scale offers a practical framework for future health and social research. Further studies are warranted to examine its performance in additional populations and its associations with relevant health outcomes.

**Supplementary Information:**

The online version contains supplementary material available at 10.1186/s12889-026-26282-y.

## Background

Socioeconomic status (SES) is a cornerstone of social epidemiology and one of the strongest determinants of health at individual, family, and community levels [1, 2]. Differences in SES influence health behaviors, disease risk, and access to healthcare services, leading to persistent health inequalities [3]. Populations with lower SES often face barriers to healthcare, inadequate nutrition, and exposure to adverse environmental conditions, resulting in poorer health outcomes [4].

Despite its importance, SES is a multidimensional construct that cannot be fully captured by a single indicator [5]. It generally encompasses domains such as education, occupation, and economic resources, which together reflect both social position and material well-being [6]. Education affects employment opportunities, health literacy, and care-seeking behavior, while occupation reflects both income potential and social prestige. Economic status, including income and accumulated wealth, represents a family’s capacity to maintain a decent standard of living and access healthcare [7]. Ownership of durable goods and household assets can also serve as stable indicators of long-term wealth, especially where income data are unreliable [8, 9].

Household and environmental conditions, such as housing quality, crowding, sanitation, and access to clean water, further reflect socioeconomic circumstances and directly influence health risks [10]. In Egypt, patterns of healthcare utilization are strongly shaped by socioeconomic differences. Out-of-pocket payments account for more than 70% of total health expenditure, placing substantial financial pressure on low- and middle-income households [11].

Efforts to measure SES in Egypt date back to the 1983, when Fahmy and El-Sherbini (1983) developed one of the first scales for health research [12]. Their tool assessed education, occupation, income, and housing conditions, which were relevant at that time. However, Egypt’s socioeconomic landscape has since changed dramatically. Inflation, currency depreciation, and shifts in educational and employment structures have limited the applicability of older scales. Revised versions have attempted to update income categories and living standards, but many remain lengthy, localized, or difficult to apply in large surveys [13, 14].

Given these transformations, there is a clear need for a concise and methodologically sound Family Socioeconomic Status Scale (FSESS) that reflects contemporary social and economic conditions in Egypt. The present study aimed to refine, update, and psychometrically validate an Arabic version of the FSESS by examining its content validity, construct validity, and internal consistency. The scale is intended to support socioeconomic measurement in health and social research across diverse community settings in Egypt, while further validation against health outcomes remains a subject for future research.

## Methods

### Study design and setting

A community-based cross-sectional descriptive validation study was conducted between January 1st and September 30th, 2025, to refine, update, and validate the Family Socioeconomic Status Scale (FSESS) for use in health research in Egypt. The study was implemented across Egypt’s four major governorates representing the country’s four main geographic regions to ensure national representativeness:

- Lower Egypt: Dakahlia.
- Upper Egypt: Qena.
- Greater Cairo: Cairo.
- Frontier Governorates: New Valley.

This design ensured inclusion of diverse socioeconomic and cultural settings spanning urban, rural, and frontier communities.

### Sampling design

A multistage stratified cluster sampling design was adopted to ensure representation from all major regions of Egypt. Stage 1: Egypt was stratified into four main regions: Greater Cairo, Lower Egypt, Upper Egypt, and the Frontier Governorates. One governorate was purposively selected from each region to reflect geographic, demographic, and socioeconomic heterogeneity while maintaining feasibility of fieldwork and availability of trained local collaborators. Accordingly, Cairo (Greater Cairo) was selected as a highly urbanized metropolitan area; Dakahlia (Lower Egypt) as a densely populated agricultural governorate; Qena (Upper Egypt) as a predominantly rural and less advantaged setting; and New Valley (Frontier Governorates) as a sparsely populated desert governorate. This selection strategy was intended to maximize contextual representativeness rather than generate nationally weighted estimates.

Stage 2: Within each governorate, three districts were randomly selected, yielding a total of 12 districts (4 governorates × 3 districts each). Stage 3: From each district, two localities were chosen—one city (district capital) and one village—to capture both urban and rural contexts. In Cairo, which is entirely urban, two different cities (urban districts) were selected instead. Stage 4: Within each selected city or village, five clusters were identified to represent the central, northern, southern, eastern, and western areas. This yielded a total of 120 clusters (4 governorates × 3 districts × 2 localities × 5 clusters = 120).

**Fig. 1:**
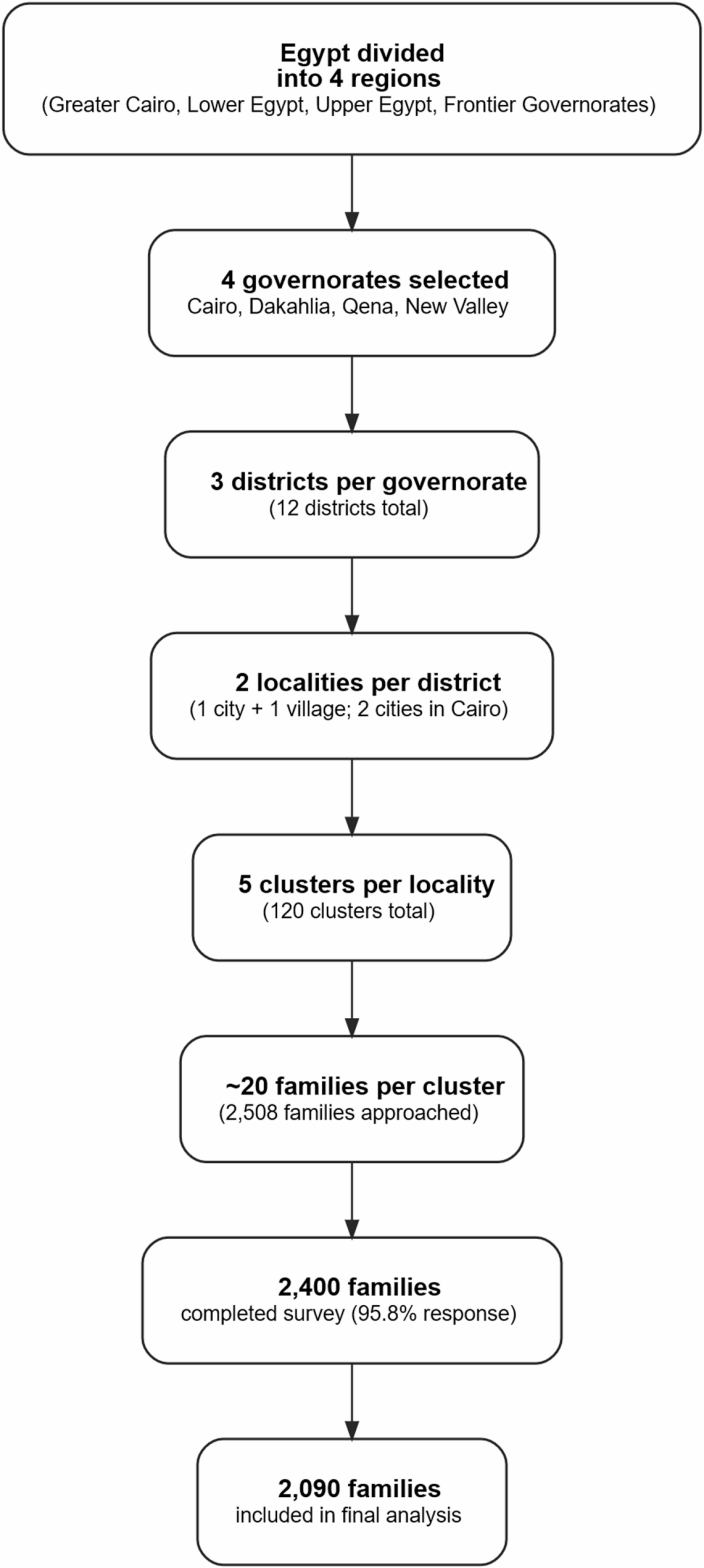
Multistage sampling flow for the refinement and validation of the Family Socioeconomic Status Scale (FSESS) across Egyptian governorates

Each cluster included approximately 20 nuclear families, giving a total of 2,400 families (120 × 20 = 2,400). The first household in each cluster was randomly selected according to predefined randomization rules. In cases of refusal or ineligibility, the next eligible household was approached as a replacement. Local primary healthcare personnel assisted field teams in identifying households from varying socioeconomic backgrounds to ensure adequate diversity within each sample area. Families were eligible for inclusion if they constituted a nuclear family (one or two parents with or without unmarried children). Extended or polygamous families and non-Egyptian households were excluded. Figure 1 illustrates the multistage sampling process used in this study, from the regional division of Egypt to the final number of families included in the analysis.

### Sample size

The required sample size was determined based on a respondent-to-item ratio of 10:1, following the guidelines of Hair Junior and colleagues (2009) and DeVellis (2003) [15, 16]. Given that the preliminary version of the Family Socioeconomic Status Scale (FSESS) consisted of twenty items, a minimum of two hundred families per district were targeted to ensure adequate statistical power for psychometric validation. A total of 2,506 families were invited to participate, of whom 2,400 completed the survey, resulting in a response rate of 95.8%. After excluding cases with incomplete or non-applicable data, particularly missing information on parental education or occupation (e.g., cases of single-parent households due to death or divorce), 2,090 families were included in the final analysis. This sample size was sufficient to perform both exploratory factor analysis (EFA) and confirmatory factor analysis (CFA) with robust reliability and validity testing.

### Data collection approach

Data were collected through face-to-face interviews conducted by trained data collectors during home visits. Interviews were administered in Arabic, ensuring clarity and cultural appropriateness.

Although interviews were conducted verbally in Arabic, responses were recorded electronically on tablet devices using a structured Google Forms questionnaire in English to facilitate standardized data entry and centralized database management. This hybrid approach minimized data entry errors and allowed real-time monitoring of data completeness by supervisors.

Each interview was conducted with either the head of household or a knowledgeable adult family member. Daily review of submissions was performed by field supervisors to ensure accuracy, consistency, and completeness before data export and cleaning.

### Training of data collectors

Data collection was conducted by medical students from participating governorates who served as field investigators. To ensure consistency in interviewing techniques and accuracy in data recording, a comprehensive training program was organized prior to fieldwork. The training included four interactive sessions delivered via Zoom, each focusing on different aspects of the study process. The first session provided an orientation and overview of the study’s objectives, methodology, and ethical principles. The second session offered a detailed explanation of the Family Socioeconomic Status Scale (FSESS), emphasizing conceptual understanding, response categories, and scoring procedures. The third session focused on interviewing techniques, highlighting the importance of neutrality, cultural sensitivity, and effective communication in Arabic. The fourth session provided technical guidance on the use of Google Forms, troubleshooting common issues, and maintaining data security and confidentiality.

To reinforce learning and ensure standardization, role-play exercises and mock interviews were conducted to simulate real field conditions. Following the training, a pilot test was implemented to evaluate the clarity and applicability of the questionnaire, as well as the interviewers’ performance. Feedback sessions were subsequently held to address questions, clarify procedures, and strengthen consistency across all data collectors before the commencement of actual data collection.

### Scale development and item generation

The revised Family Socioeconomic Status Scale (FSESS) was adapted from El-Gilany and colleagues in 2012 and previous Egyptian and international socioeconomic scales [1, 8, 9, 17, 18]. The item pool was developed through an extensive literature review and online expert panel discussions that included five public health experts and one sociologist (*n* = 6).

Twenty candidate items were initially identified, covering education, occupation, family income, possessions, housing, social participation, and healthcare access. Occupational categories were based on the Unified Occupational Classification Guide [19].

The initial 20 items included:

- 1-2. Husband’s and wife’s education
- 3-4. Husband’s and wife’s occupation
- 5-8. Job contract and work pattern (for husband and wife)
- 9. Family possessions and assets
- 10. Family monthly income (all sources)
- 11. Governmental support
- 12-14. Family residence, family size, and number of earners
- 15-17. Home sanitation, housing type, and crowding index
- 18. Social and cultural power
- 19. Access to health information
- 20. Usual source of healthcare

Each item included predefined categorical responses with corresponding scores (Full scoring details are available in the Supplementary file 1).

### Content validity and translation procedures

The initial item pool of the Family Socioeconomic Status Scale (FSESS) was developed in English following an extensive review of Egyptian and international socioeconomic measurement literature. To ensure linguistic and conceptual equivalence for field application, the scale was subsequently translated into Arabic using a forward and backward translation process.

First, the English version was independently translated into Arabic by bilingual researchers with public health backgrounds. The Arabic version was then back-translated into English by an independent bilingual translator who was blinded to the original version. Discrepancies between the original and back-translated English versions were reviewed and resolved through consensus discussions, with emphasis on conceptual rather than literal equivalence.

Content validity was then assessed using both the English and Arabic versions of the scale. Thirteen independent public health experts evaluated each item for relevance and clarity using a 3-point ordinal scale. Item-level Content Validity Index (I-CVI), Expert-level Content Validity Index (E-CVI), and Scale-level Content Validity Index (S-CVI) were calculated in accordance with established guideline [20, 21]. Items with an I-CVI of 0.79 or higher were retained, and minor wording and scoring adjustments were made based on expert feedback to enhance clarity, cultural appropriateness, and consistency across both language versions. This iterative bilingual development process ensured that the Arabic interview version and the English electronic data-entry version were conceptually aligned and psychometrically equivalent.

### Reliability testing

Internal consistency reliability of the Family Socioeconomic Status Scale (FSESS) was assessed using Cronbach’s alpha coefficient. Cronbach’s alpha was calculated for the overall scale and examined at the item level using the “alpha if item deleted” approach to evaluate the contribution of individual items to scale coherence. Alpha values of 0.50–0.70 were considered acceptable, and values ≥ 0.70 were considered indicative of good internal consistency, in line with established psychometric guidelines [22].

### Construct validity

#### Exploratory factor analysis (EFA)

EFA was conducted using the full dataset of 2,090 families to identify the underlying structure of the Family Socioeconomic Status Scale (FSESS). The analysis employed Principal Component Analysis (PCA) with varimax rotation. Data adequacy was confirmed by a Kaiser–Meyer–Olkin (KMO) value of 0.89 and a significant Bartlett’s test of sphericity (*p* < 0.001), indicating suitability for factor analysis. Items with factor loadings ≥ 0.60 and no significant cross-loading were retained. Three interpretable factors emerged, Education, Occupation, and Family income and possessions, which collectively explained 49% of the total variance.

### Confirmatory factor analysis (CFA)

Subsequently, CFA was performed on the same dataset (*n* = 2,090) using R version 4.3.3 with maximum likelihood estimation to verify the factor structure identified in EFA. Using the same sample is acceptable in this context because of the large sample size and the exploratory–confirmatory sequence applied within a single validation study. Model fit was assessed using multiple goodness-of-fit indices, which demonstrated satisfactory results (χ²/df < 5, RMSEA < 0.08, GFI, CFI, and IFI > 0.90). All standardized factor loadings were statistically significant (*p* < 0.001), confirming the scale’s construct validity and robustness of the final three-factor model.

Although independent samples are ideal, the use of the same large sample for both EFA and CFA is considered acceptable in scale development studies when sample size is substantial and factor structure is stable, as this approach yields reliable parameter estimates and model convergence.

Although the use of independent samples for exploratory and confirmatory factor analyses is methodologically ideal, this approach is not always feasible in large-scale field studies. Several methodological authorities indicate that conducting EFA and CFA on the same dataset is acceptable when the sample size is sufficiently large and the resulting factor structure is stable and theoretically coherent [23]. In the present study, the large sample size (*n* = 2,090), high communalities, and consistently strong factor loadings across analyses reduce the risk of overfitting and support the robustness of the identified three-factor model. This approach has been commonly adopted in scale development and validation studies under similar conditions and is considered to yield reliable parameter estimates and model convergence.

### Scoring system

Following factor analysis, six core items were retained in the final validated version of the Family Socioeconomic Status Scale (FSESS), with a maximum total score of 45 points. In cases where information for one parent was unavailable (e.g., due to death, divorce, or absence), the score was recalculated based on a maximum of 29 points to maintain proportional comparability.

To facilitate interpretation, a normalized socioeconomic status (SES) percentage was calculated using the following formula:

$$\:\mathrm{S}\mathrm{E}\mathrm{S}\:\mathrm{s}\mathrm{c}\mathrm{o}\mathrm{r}\mathrm{e}=100\:\times\:\:\frac{\mathrm{R}\mathrm{a}\mathrm{w}\:\mathrm{S}\mathrm{c}\mathrm{o}\mathrm{r}\mathrm{e}}{\mathrm{M}\mathrm{a}\mathrm{x}\mathrm{i}\mathrm{m}\mathrm{u}\mathrm{m}\:\mathrm{P}\mathrm{o}\mathrm{s}\mathrm{s}\mathrm{i}\mathrm{b}\mathrm{l}\mathrm{e}\:\mathrm{S}\mathrm{c}\mathrm{o}\mathrm{r}\mathrm{e}}$$

This normalization approach allows for standardization of scores across different family structures.

While CFA and SEM were used to validate the latent structure and relative contribution of indicators, the final FSESS scoring system retains a simplified additive format to ensure feasibility in large-scale field surveys and routine health research. This operational scoring approach is consistent with common practice in applied socioeconomic measurement.

### Example

If a family achieved a raw score of 30 out of a maximum 45, their normalized SES percentage would be:

$$\:100\:\times\:\:\frac{30}{45}=66.7\mathrm{\%}$$

This score would classify the family within the middle socioeconomic class.

Based on normalized SES percentages, families were categorized into four classes:

- Very low: 1–25%.
- Low: 26–50%.
- Middle: 51–75%.
- High: ≥76%.

To facilitate the practical application of the validated Family Socioeconomic Status Scale (FSESS), we developed an open-access online calculator available at: https://mohamedterra.github.io/FSESS_Calculator/. This tool enables researchers and practitioners to easily compute SES scores and classifications based on the refined FSESS model.

### Statistical analysis

All statistical analyses were performed using IBM SPSS Statistics version 29.0 (IBM Corp., Armonk, NY), and R software version 4.3.3. Descriptive statistics, including means, standard deviations, frequencies, and percentages, were used to summarize participants’ sociodemographic characteristics and responses to the FSESS items. Reliability was evaluated using Cronbach’s alpha coefficient to assess internal consistency. Content validity indices (CVI) were calculated for individual items, experts, and the overall scale.

Exploratory factor analysis (EFA) was conducted using principal component analysis with varimax rotation to identify the underlying factor structure of the scale. The suitability of data for factor analysis was examined using the Kaiser–Meyer–Olkin (KMO) measure of sampling adequacy and Bartlett’s test of sphericity. Confirmatory factor analysis (CFA) was subsequently performed to validate the extracted factor structure, employing maximum likelihood estimation in R. Model fit was assessed using multiple indices, including the chi-square to degrees of freedom ratio (χ²/df), the Comparative Fit Index (CFI), the Incremental Fit Index (IFI), the Goodness-of-Fit Index (GFI), and the Root Mean Square Error of Approximation (RMSEA) with a 90% confidence interval. Acceptable model fit was indicated by χ²/df < 5, RMSEA < 0.08, and GFI, CFI, and IFI values above 0.90.

Structural equation modeling (SEM) was also applied to explore the interrelationships among the latent constructs of education, occupation, and family income and possessions. Statistical significance was set at *p* < 0.05 for all analyses.

## Results

### Sample characteristics

The study included 2,400 families from different governorates across Egypt. The detailed sociodemographic characteristics of the participating households are presented in Supplementary file 2. Notably, the most common educational level for both husbands (36.0%) and wives (36.9%) was secondary education, followed by university education among 24.5% of husbands and 30.6% of wives. A smaller proportion of husbands (6.0%) and wives (5.0%) had postgraduate qualifications. Occupationally, the majority of wives (60.2%) were housewives, whereas husbands demonstrated more occupational diversity, with the largest categories being skilled manual workers (25.4%) and professionals (23.2%). Approximately 68.2% of husbands held a permanent work contract, compared to 29.5% of wives. With respect to family income and possessions and living conditions, almost all families owned at least one refrigerator (98.1%), and a high proportion possessed smartphones (94.1%) and washing machines (64.1%). Ownership of a car (42.0%) and computers (59.1%) indicated a generally moderate-to-high level of material assets.

Regarding income sufficiency, 37.2% of families reported that their income “meets expenses and emergencies,” and 30.1% were “able to save and invest,” while 28.0% indicated their income “just meets expenses.” Nearly half of households (49.1%) reported receiving governmental support through subsidized food programs. In terms of residence, half of the families (50.0%) lived in rural areas, 37.5% in urban settings, and 12.5% in slum areas. The average family size was moderate, with 65.5% of households having five to six members. Most families (51.9%) had two earning members, and 18.0% reported more than two. Housing conditions reflected satisfactory sanitation infrastructure, with 98.2% having access to electricity, 92.0% to a sewerage system, and 96.7% to safe drinking water. The crowding index indicated that 28.7% of families had one or fewer persons per room.

### Content validity

Item- and expert-level Content Validity Indices (CVIs) are summarized in Table 1. The item-level CVI (I-CVI) for relevance ranged from 0.69 to 1.0, with a mean value of 0.87, indicating strong expert agreement on item relevance. The I-CVI for clarity ranged from 0.54 to 0.92 (mean = 0.77), suggesting acceptable linguistic and conceptual clarity of items. At the expert level, the E-CVI for relevance ranged between 0.60 and 1.00, and the E-CVI for clarity ranged from 0.60 to 1.00, confirming high inter-expert consistency and strong overall content validity of the developed socioeconomic scale. These findings affirm that the items comprehensively represented the construct intended to measure socioeconomic status among Egyptian families.

**Table 1:** Item and expert content validity indices (CVIs) of different items of the scale

Item	Relevance I-CVI	Clarity I-CVI	Expert	Relevance E-CVI	Clarity E-CVI
Husband education	0.92	0.85	Expert 1	0.60	0.65
Wife education	0.92	0.85	Expert 2	0.95	1.00
Husband job	0.92	0.77	Expert 3	0.60	0.90
Wife job	0.92	0.77	Expert 4	0.60	1.00
Husband work contract	0.85	0.69	Expert 5	0.95	0.90
Wife work contract	0.85	0.69	Expert 6	1.00	1.00
Husband working hours	0.77	0.77	Expert 7	1.00	0.60
Wife working hours	0.77	0.77	Expert 8	0.65	1.00
Family assets/possessions	0.85	0.77	Expert 9	0.90	0.95
Family income (all sources/persons)	1.00	0.85	Expert 10	0.95	1.00
Receives governmental support	0.69	0.77	Expert 11	0.95	0.85
Residence	0.92	0.69	Expert 12	0.95	0.90
Family size	0.92	0.69	Expert 13	1.00	1.00
Earning family member	0.85	0.69			
Housing sanitation	0.85	0.92			
Type of house	0.92	0.92			
Crowding index	0.92	0.92			
Social power	0.69	0.54			
Health information access	0.85	0.69			
Usual source of health care	0.92	0.69			
Average CVI	**0.87**	**0.77**			

### Reliability analysis

The internal consistency of the proposed scale was examined using Cronbach’s alpha (Table 2). The total scale achieved an alpha of 0.82, indicating good internal reliability. The “alpha if item deleted” values ranged narrowly between 0.79 and 0.82, implying that no single item significantly reduced or enhanced the scale’s reliability. Thus, all items contributed meaningfully to the overall construct. These results support the scale’s stability and internal coherence in assessing family socioeconomic conditions.

**Table 2:** Reliability of the proposed scale (Cronbach’s alpha of total scale and if item deleted)

Item	Cronbach alpha if item deleted
Husband education	0.79
Wife education	0.79
Husband job	0.79
Wife job	0.8
Husband work contract	0.82
Wife work contract	0.81
Husband working hours	0.82
Wife working hours	0.81
Family assets/possessions	0.8
Family income from all sources & persons.	0.81
Receives governmental support	0.82
Residence	0.81
Family size	0.82
Earning family member	0.82
Housing sanitation	0.81
Type of house	0.82
Crowding index	0.82
Social power	0.82
Health information access	0.82
Usual source of health care	0.81

Total scale Cronbach alpha = 0.82

### Exploratory factor analysis (EFA)

**Fig. 2:**
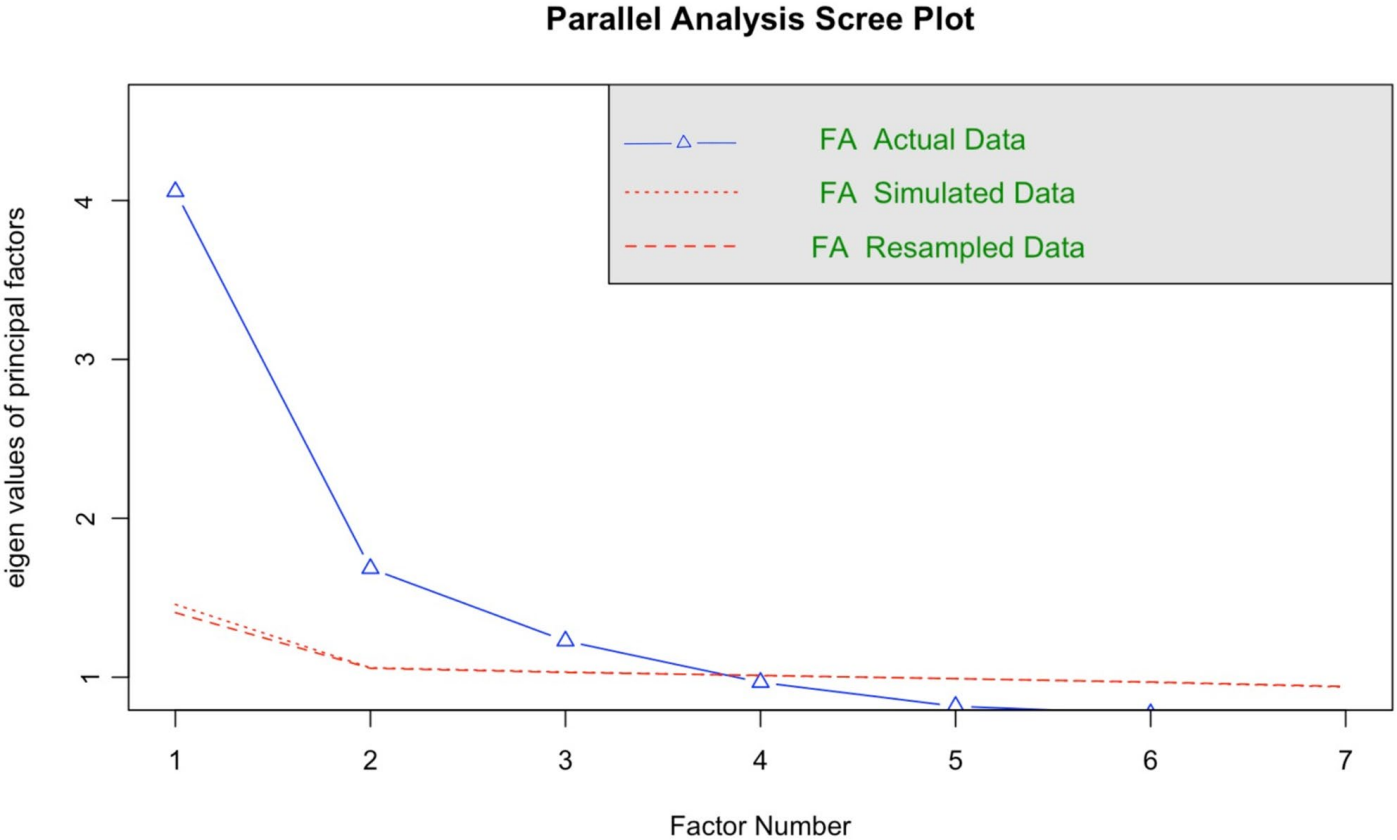
Scree plot from Exploratory Factor Analysis (EFA)

The suitability of the dataset for factor analysis was confirmed by a Kaiser–Meyer–Olkin (KMO) measure of 0.89, exceeding the recommended threshold of 0.6, and a significant Bartlett’s Test of Sphericity (*p* < 0.001). Parallel analysis and the scree plot (Fig. 2) revealed that the first three factors had eigenvalues greater than one, after which the curve leveled off, suggesting a three-factor solution. These three factors jointly accounted for the majority of the variance and were theoretically interpreted as representing Education, Occupation, and Family income and possessions. Items corresponding to educational attainment (husband’s and wife’s education), occupational characteristics (husband’s and wife’s jobs), and family financial indicators (income and possessions) loaded strongly on their respective factors, validating the conceptual framework of the scale.

### Confirmatory factor analysis (CFA)

A Confirmatory Factor Analysis (CFA) was performed to validate the three-factor structure identified through Exploratory Factor Analysis (EFA). As presented in Table 3, all standardized factor loadings were statistically significant (*p* < 0.001), ranging from 0.563 to 0.915, which demonstrates strong associations between the observed variables and their respective latent constructs.

In the CFA specification, all cross-loadings were fixed to zero, and each observed indicator was constrained to load exclusively on its hypothesized latent construct. Parsimony-adjusted fit indices further supported model adequacy (PNFI = 0.63; PCFI = 0.67), indicating a good balance between model fit and model complexity. A residual covariance between conceptually related occupational indicators was added based on theoretical considerations reflecting shared variance not captured by the latent construct. Inclusion of this covariance resulted in a significant improvement in model fit (Δχ² = 41.8, Δdf = 1, *p* < 0.001). Convergent validity was supported for all latent constructs, with average variance extracted (AVE) values ≥ 0.50 and composite reliability coefficients exceeding 0.70 (Supplementary file 3). The family possessions indicator was derived from a checklist of common household assets. The full list of items and their endorsement frequencies is presented in Supplementary file 3. No additional post-hoc modifications were introduced beyond the theoretically justified covariance specified a priori.

**Table 3:** Confirmatory factor analysis (CFA) and structural equation modeling (SEM) results

Latent Construct / Path	Indicator	Standardized Estimate (β)	*p*-value
Factor Loadings (CFA)			
Education	Husband’s education	0.915	< 0.001*
	Wife’s education	0.824	< 0.001*
Occupation	Husband’s job	0.772	< 0.001*
	Wife’s job	0.563	< 0.001*
Family income and possessions	Family income	0.635	< 0.001*
	Family possessions	0.728	< 0.001*
Structural Regression Paths (SEM)			
Jobs ← Education	—	0.850	< 0.001*
Family income and possessions ← Education	—	–0.013	0.894
Family income and possessions ← Jobs	—	0.746	< 0.001*
Covariances among Latent Variables			
Education ↔ Jobs/Work	—	0.850	< 0.001*
Education ↔ Family income and possessions	—	0.621	< 0.001*
Jobs/Work ↔ Family income and possessions	—	0.735	< 0.001*

*p < 0.05 indicates statistical significance

The Education factor showed high loadings for husband’s education (β = 0.915) and wife’s education (β = 0.824). The Occupation factor was represented by wife’s job (β = 0.563) and husband’s job (β = 0.772). The Family income and possessions factor comprised family income (β = 0.635) and family possessions (β = 0.728).

### Structural equation modeling (SEM)

**Fig. 3:**
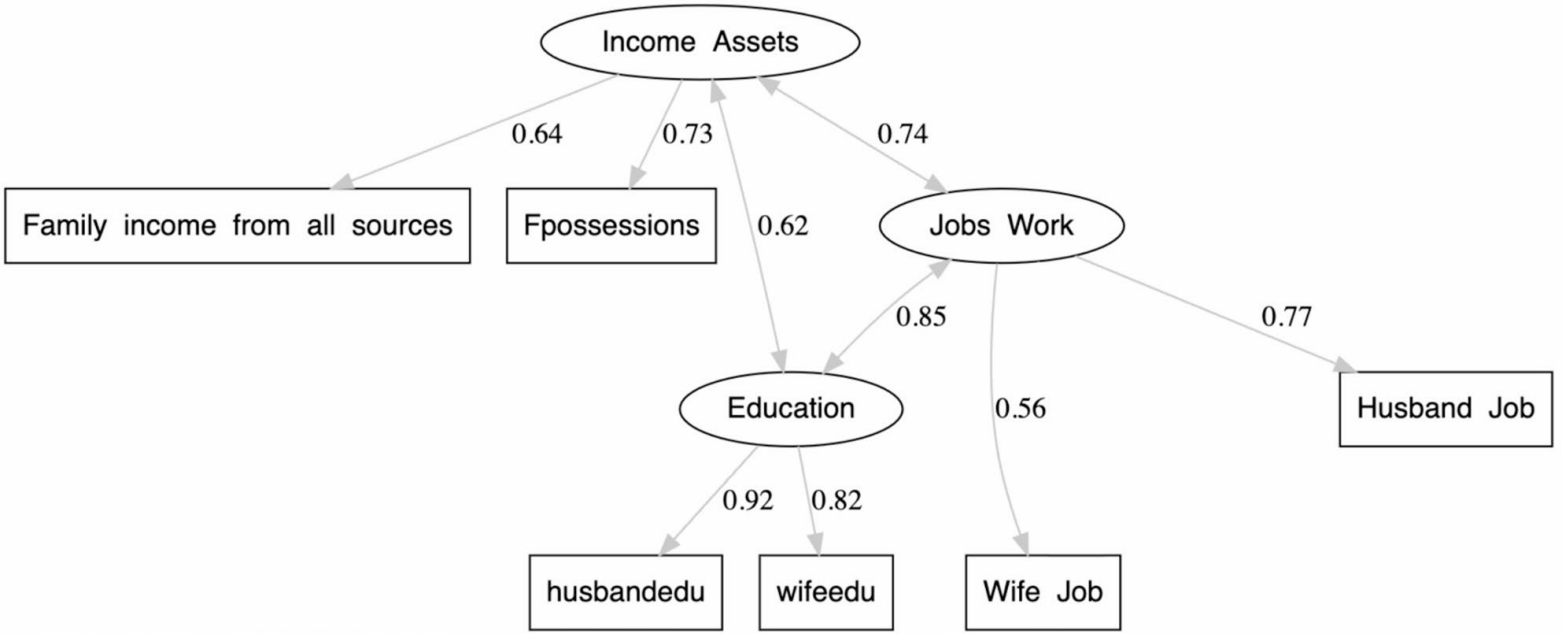
SEM path diagram showing standardized regression coefficients and covariances

To further assess the interrelationships among the latent constructs, a Structural Equation Model (SEM) was estimated (Fig. 3; Table 3). The model demonstrated an excellent fit to the data.

(χ² = 8826.15, df = 21, *p* < 0.001; CFI = 0.95; TLI = 0.94; RMSEA = 0.045, 90% CI 0.038–0.052; SRMR = 0.037). As shown, Education was strongly associated with Jobs/Work (β = 0.85, *p* < 0.001), indicating that higher educational attainment is strongly associated with improved occupational outcomes. In turn, Jobs/Work significantly predicted Income/Assets (β = 0.75, *p* < 0.001), suggesting that employment quality and stability mediate the link between education and household economic resources. The direct path from Education to Income/Assets was not significant (β = − 0.013, *p* = 0.894), confirming full mediation through occupational status.

Covariance analysis revealed significant positive correlations among all latent constructs. Education was strongly correlated with Jobs/Work (*r* = 0.85, *p* < 0.001) and moderately correlated with Income/Assets (*r* = 0.62, *p* < 0.001). Similarly, a strong positive association was observed between Jobs/Work and Income/Assets (*r* = 0.74, *p* < 0.001). These relationships indicate a coherent structural pattern in which higher educational attainment enhances occupational opportunities, which, in turn, lead to improved family income and asset ownership.

## Discussion

Socioeconomic status (SES) is a multidimensional construct that reflects access to financial, social, cultural, and human resources and plays a central role in shaping population health and wellbeing. The Family Socioeconomic Status Scale (FSES) provides a structured and standardized approach to capturing these dimensions. Although SES is not a medical construct, it is widely used in public health research to describe social stratification and contextual factors that influence health patterns and access to services [4]. Careful selection of SES indicators is therefore essential to ensure conceptual clarity and psychometric validity [24].

Previous studies have highlighted that there is little consensus regarding the optimal indicators of SES for health research [25]. Each dimension of SES captures a distinct aspect of social stratification, and no single indicator can provide a complete picture. Egypt has undergone significant socioeconomic transformation, particularly after the 2011 revolution, which was accompanied by rising unemployment, increasing poverty, and widening income disparities [26]. Given this dynamic context and the ongoing inflationary pressures, earlier SES scales that relied heavily on fixed income measures became less accurate and required modification to remain relevant [13, 27, 28].

The present revision of the FSES aimed to simplify and update existing Egyptian scales by focusing on indicators that remain informative under changing economic conditions. Measuring SES is inherently complex and often relies on proxy indicators to capture underlying social structures. Composite analytic techniques, such as principal component analysis, are therefore well suited to identify key dimensions. Consistent with previous research [7, 29], the findings of this study support education, occupation, and family income and possessions as the most stable and informative dimensions of family socioeconomic position within the studied settings. These dimensions capture complementary aspects of SES and are not assumed to be interchangeable, as educational attainment, occupational status, and material resources may follow different trajectories across the life course [30, 31].

The refined FSES includes three core latent constructs: parental education, parental occupation, and family income and possessions, including income and possessions. These were identified as the strongest indicators through confirmatory factor analysis and structural equation modeling, aligning with findings by Caro and Cortés (2012) [32]. Variables such as residence, family size, sanitation, and healthcare source demonstrated lower factor loadings, suggesting that they contribute less to the current socioeconomic differentiation across Egyptian families, which supports the findings of El-Gilany et al. (2012) [33].

Education remains one of the most accessible and stable SES indicators. It provides individuals with cognitive skills, health literacy, and opportunities for stable employment. Yet, it has notable limitations. It may not fully capture current wealth or informal skills and may lose its discriminatory power as education levels rise nationally [24]. The socioeconomic meaning of education also varies by time and context, as the returns to education depend on labor market conditions and the structure of the economy [34].

Occupation, separated from education in this updated scale, captures dimensions such as income potential, work stability, and exposure to occupational hazards. In modern economies, it is not uncommon for skilled trades to provide higher income than some professional positions, making occupation an independent determinant of SES [35]. Nevertheless, occupation cannot be applied to all individuals, such as homemakers, students, or retirees, and it is affected by gender norms and changes in labor markets [34, 35].

Income is often considered a direct indicator of economic capacity, but its measurement is challenging, particularly in countries like Egypt where informal employment is widespread. In this study, income was assessed relative to household expenses to address variability in living costs and seasonal income fluctuations, particularly among rural workers. Reported income can be unstable, influenced by family size, and prone to underreporting due to social sensitivity [36]. It also fluctuates with life stages, increasing during mid-adulthood and declining after retirement. Recent research from Bangladesh has shown that income levels are influenced not only by education and occupation but also by social networks and community participation [37].

Family income and possessions provides a more stable measure of socioeconomic position than income alone, reflecting accumulated resources and long-term economic resilience. It encompasses both tangible and intangible assets that buffer families against financial shocks and support intergenerational stability [38, 39]. Wealth-related measures may show stronger associations with health outcomes, particularly in older adults who rely on accumulated assets rather than active income [38]. However, collecting detailed wealth data can be difficult, as respondents may lack documentation or be reluctant to disclose their assets.

Composite SES scales, such as the revised FSES, offer an integrative approach that combines multiple indicators into a single measure. This approach enhances comparability, reduces respondent burden, and simplifies analysis [5]. Nevertheless, assigning relative weights to each component introduces some subjectivity, and composite measures may obscure the unique contributions of specific variables to health outcomes [40].

The limited contribution of housing sanitation and utilities in the present study likely reflects improvements in basic infrastructure resulting from recent national development initiatives, including the “Decent Life” program [41]. Similarly, residential location alone may not adequately represent socioeconomic position in large urban areas where households of different economic levels coexist within the same neighborhoods [42].

The usual source of healthcare was not retained in the final model, which may be explained by recent health system reforms in Egypt that have expanded access to essential services across population groups [43]. While healthcare utilization continues to be influenced by social and cultural factors [44], its reduced variability limits its usefulness as a differentiating SES indicator in the current context.

From a practical perspective, the revised FSES offers a feasible tool for use in population-based surveys, health research, and social monitoring systems in Egypt. Its concise structure makes it suitable for large-scale data collection and program evaluation, particularly in assessing socioeconomic inequalities and their implications for health and wellbeing. Taken together, the revised FSES provides a concise and culturally informed framework for assessing family socioeconomic position in Egypt. By reducing the scale to six core indicators, it balances feasibility with conceptual rigor. While the present study focused on psychometric refinement, further validation against health outcomes and in additional population groups is warranted to strengthen its application in public health and social research.

### Limitations

This study has several limitations that should be considered when interpreting the findings. First, the validation was restricted to nuclear families consisting of parents and their unmarried children, which may limit applicability to extended or polygamous households that remain present in some Egyptian communities. Adaptation of the scale for these family structures will require additional methodological work and field testing. Second, key socioeconomic indicators such as income sufficiency and household possessions were self-reported, which may be subject to recall bias or social desirability bias, particularly in settings where financial disclosure is sensitive. Although the inclusion of asset-based indicators was intended to improve stability compared with income alone, some degree of reporting error cannot be excluded.

Third, the study focused primarily on content validity, construct validity, and internal consistency, and did not assess criterion or concurrent validity against specific health outcomes. Socioeconomic status does not have a single health-based gold standard, and health outcomes are influenced by complex interactions among biological, behavioral, environmental, and social factors. Consequently, no single health indicator can adequately validate an SES construct. Nevertheless, future studies should evaluate the predictive and concurrent validity of the Family Socioeconomic Status Scale in relation to relevant health outcomes using outcome-oriented or longitudinal designs. Fourth, although the sample included families from four governorates representing major geographic regions of Egypt, the findings may not fully capture socioeconomic variation in informal urban settlements, remote frontier communities, or highly mobile populations. Further validation in these settings is recommended before broader generalization.

Fifth, exploratory and confirmatory factor analyses were conducted within the same dataset. While this approach is considered acceptable in scale development studies with large samples and stable factor structures, independent replication in separate samples would further strengthen external validity. Finally, the cross-sectional design of the validation process did not allow assessment of temporal stability or test–retest reliability. Longitudinal studies are therefore needed to evaluate the stability and responsiveness of the scale over time.

## Conclusion

This study refined and validated a concise Family Socioeconomic Status Scale (FSES) designed to support socioeconomic measurement in health and social research contexts in Egypt. The final scale identified education, occupation, and family income and possessions as the most stable and informative indicators of family socioeconomic position within the studied settings. By reducing the original item pool to a small number of core indicators, the revised FSES offers a practical and methodologically sound tool that is feasible for use in large population-based surveys.

The scale was developed in response to ongoing social and economic changes in Egypt, including inflation, shifts in educational attainment, and labor market transformations that have reduced the usefulness of traditional income-only measures. Rather than relying on a single economic indicator, the FSES captures multiple dimensions of socioeconomic position while remaining simple to administer and interpret. As such, the FSES can be readily incorporated into national health surveys, epidemiological studies, and social monitoring systems to assess socioeconomic inequalities and their relationship with health outcomes. It may also be applied in program evaluation to examine whether public health and social interventions effectively reach and benefit disadvantaged families. In addition, the concise structure of the scale makes it suitable for routine data collection and longitudinal monitoring of socioeconomic trends in Egypt.

Although the scale was validated across geographically diverse governorates, its findings should be interpreted within the context of the study design and sample. The present work focused on psychometric refinement and construct validation and did not evaluate associations with specific health outcomes. Future research should therefore assess the predictive and concurrent validity of the FSES in relation to relevant health indicators, examine its performance in extended and polygamous family structures, and confirm its stability through longitudinal designs. Overall, the refined FSES provides a transparent and adaptable measurement framework that can facilitate future investigations of socioeconomic gradients in health and support evidence-informed public health and social research in Egypt.

## Supplementary Information

Supplementary Material 1.

Supplementary Material 2.

Supplementary Material 3.

## Data Availability

The datasets used and/or analyzed during the current study are available from the corresponding author upon reasonable request.
