# Supplementary material for: Refining and validation of Family Socioeconomic Status Scale (FSESS) for health research in Egypt
Source: BMC Public Health. 2026 Feb 9;26:728. doi: 10.1186/s12889-026-26282-y (PMC12930555; doi:10.1186/s12889-026-26282-y)
Supplement: Supplementary file 1 — Supplementary Material 1. [file 12889_2026_26282_MOESM1_ESM.docx]

**Supplementary file 1.** Final Short Form of the Family Socioeconomic Status Scale (FSES) and Its Scoring (English–Arabic)

| **Item / البند** | **Response categories / الفئات** | **Score (Husband / الزوج)** | **Score (Wife / الزوجة)** |
| --- | --- | --- | --- |
| Education / التعليم | Non-applicable (death/divorce) / لا ينطبق (الوفاة أو الطلاق)  Illiterate / أمي  Less than secondary / أقل من ثانوي  Secondary / ثانوي  University / جامعي  Postgraduate / دراسات عليا | 0  2  4  6  8  10 | 0  2  4  6  8  10 |
| Occupation / المهنة | Non-applicable (death/divorce) / لا ينطبق (الوفاة أو الطلاق)  Non-working / Housewife / لا يعمل – ربة منزل  Unskilled manual worker / عامل غير ماهر  Skilled manual worker / عامل ماهر  Army or police (soldiers & NCOs) / جنود وصف ضباط الجيش والشرطة  Business / Trade / العاملون في التجارة أو الأعمال الحرة  Clerical / Office worker / الكتبة وموظفو المكاتب  Professional / Legislative / Director / Commissioned officer / أصحاب المهن العلمية والمشرعون والمديرون وضباط الجيش والشرطة | 0  0  1  2  3  4  5  6 | 0  0  1  2  3  4  5  6 |
| Family Income / دخل الأسرة من جميع المصادر | Indebt / يستدين  Barely meets basic needs / يكفي الأساسيات بالكاد  Meets needs + emergencies / يكفي الأساسيات والطوارئ  Saves and invests / يدخر ويستثمر | 0  1  2  3 | |
| Family Assets & Possessions / ممتلكات الأسرة | **One point for each of the following (maximum = 10):** Refrigerator / ثلاجة  Automatic washing machine / غسالة أوتوماتيك  Smartphone / هاتف ذكي  Air conditioner / مكيف هواء  Agricultural land / أرض زراعية  Land for housing / أرض للمباني  Shop / Animal farm / محل تجاري / مزرعة مواشي  Another house / منزل آخر  Car / سيارة  Computer / كمبيوتر | Up to 10 points | |

**Total score / المجموع الكلي:** Maximum possible score = **45 points** (or **29 points** if certain items are not applicable).

الحد الأقصى للمجموع الكلي = 45 درجة (أو 29 درجة في حالة وجود بنود غير منطبقة).

**Additional Notes / ملاحظات إضافية**

- If more than one job is reported, use the occupation with the highest score.
  في حالة وجود أكثر من وظيفة، يتم اعتماد الوظيفة ذات الدرجة الأعلى.
- **Occupation categories / تصنيفات المهنة:**
  - 0 = No job (students, retirees, housewives) / لا يعمل (طلاب، متقاعدون، ربات منازل)
  - 1 = Unskilled workers (e.g., street vendors, guards, farmers, porters, servants) / عاملون غير مهرة (بائعون، حراس، مزارعون، عمال خدمات)
  - 2 = Skilled workers (e.g., drivers, carpenters, electricians, mechanics, small business owners) / عاملون مهرة (سائقون، نجارون، كهربائيون، أصحاب مشروعات صغيرة)
  - 3 = Army & police (non-officers) / عسكريون دون رتبة ضابط
  - 4 = Business & sales / تجارة وأعمال حرة
  - 5 = Clerical / office workers / موظفون إداريون ومكتبيون
  - 6 = Professionals (physicians, teachers, lawyers, engineers, officers) / أصحاب المهن العلمية (أطباء، معلمون، محامون، مهندسون، ضباط)
- **SES classification (based on total % score):**
  - 1–25% = Very Low / منخفض جدًا
  - 26–50% = Low / منخفض
  - 51–75% = Middle / متوسط
  - ≥76% = High / مرتفع
