# Supplementary material for: Refining and validation of Family Socioeconomic Status Scale (FSESS) for health research in Egypt
Source: BMC Public Health. 2026 Feb 9;26:728. doi: 10.1186/s12889-026-26282-y (PMC12930555; doi:10.1186/s12889-026-26282-y)
Supplement: Supplementary file 2 — Supplementary Material 2. [file 12889_2026_26282_MOESM2_ESM.docx]

**Supplementary table:** Descriptive statistics of different parameters of 2400 studied families

| **Variable** | **score** | **N (%)** |
| --- | --- | --- |
| Husband education: Non-applicable  Illiterate  Less than secondary  Secondary  University  Post graduate | 0  2  4  6  8  10 | 237 (9.9)  190 (7.9)  377 (15.7)  863 (36.0)  589 (24.5)  144 (6.0) |
| Wife education: Non-applicable  Illiterate  Less than secondary  Secondary  University  Post graduate | 0  2  4  6  8  10 | 115 (4.8)  248 (10.3)  295 (12.3)  886 (36.9)  735 (30.6)  121 (5.0) |
| Husband job*: Non-applicable  Non-working  Unskilled manual workers  Skilled manual workers  Army/police solders &NCO  Business/trades  Clerks/offices workers  Professional | 0  0  1  2  3  4  5  6 | 209 (8.7)  133 (5.4)  284 (11.8)  610 (25.4)  29 (1.2)  294 (12.3)  284 (11.8)  557 (23.2) |
| Wife job*: Non-applicable  Housewives  Unskilled manual workers  Skilled manual workers  Army/police solders &NCO  Business/trades  Clerks/offices workers  Professional | 0  0  1  2  3  4  5  6 | 101 (4.2)  1445 (60.2)  76 (3.2)  56 (2.3)  3 (0.1)  27 (1.1)  210 (8.8)  484 (20.1) |
| Husband work contract: Non-applicable  Non-working  Temporary contract  Permanent contract | 0  0  1  2 | 209 (8.7)  133 (5.4)  421 (17.5)  1637 (68.2) |
| Husband work hours: Non-applicable  Non-working  Part-time  Full-time | 0  0  1  2 | 209 (8.7)  133 (5.4)  456 (19.0)  1602 (66.8) |
| Wife work contract: Non-applicable  Housewives  Temporary contract  Permanent contract | 0  0  1  2 | 101 (4.2)  1445 (60.2)  146 (6.1)  708 (29.5) |
| Husband work hours: Non-applicable  Houswives  Part-time  Full-time | 0  0  1  2 | 101 (4.2)  1445 (60.2)  170 (7.1)  684 (28.5) |
| Family assets & possessions: Refrigerator  Automatic washing machine  Smart phone  Air condition  Agricultural land  Land for housing  Shops/animals  Another house  Car  Computer | 1  1  1  1  1  1  1  1  1  1 | 2354 (98.1)  1538 (64.1)  2258 (94.1)  1030 (42.9)  870 (36.0)  584 (24.3)  666 (27.8)  630 (26.3)  1008 (42.0)  1418 (59.1) |
| Family income from all sources: Indebt  Just meet expenses  Meet expenses and emergencies  Save and invest | 0  1  2  3 | 113 (4.7)  672 (28.0)  892 (37.2)  723 (30.1) |
| Family receives governmental support (subsidized food) | 1 | 1178 (49.1) |
| Residence: Slum  Rural  Urban | 0  1  2 | 300 (12.5)  1200 (50.0)  900 (37.5) |
| Family size: >6 persons  5 & 6 persons  < 5 persons | 0  1  2 | 93 (3.9)  1572 (65.5)  735 (30.6) |
| Earning family member: one  Two  More than two | 0  1  2 | 721 (30.0)  1246 (51.9)  433 (18.0) |
| Home sanitations: Pure water  Electricity  Natural gas  Sewerage system  Solid waste collection  Flush latrine | 1  1  1  1  1  1 | 2308 (96.7)  2356 (98.2)  1015 (42.3)  2208 (92.0)  1771 (73.0)  1889 (78.7) |
| Type of house: Hut/tent  Rented <5 rooms  Rented 5 rooms or more  Owned <5 rooms  Owned 5 rooms or more | 0  1  2  3  4 | 8 (0.3)  315 (13.1)  121 (5.0)  1336 (55.7)  620 (25.0) |
| Home crowding index: > 2person/room  2 to >1 person/room  1 or less/room | 0  1  2 | 138 (5.8)  1572 (65.5)  690 (28.7) |
| Social power: Social and sport club membership  Political parties’ membership  Non-governmental organization membership  Head of a tribe or extended family  Home library  Religious/social leader | 1  1  1  1  1  1 | 486 (20.3)  166 (6.9)  420 (17.5)  140 (5.8)  221 (9.2)  182 (7.6) |
| Health information access | 1 | 1951 (81.3) |
| Usual source of health care: Traditional/self-care  Free governmental services  Health insurance  Private | 0  1  2  3 | 101 (4.2)  732 (30.5)  475 (19.8)  1092 (45.5) |

NCO=nom-commissioned officers

*** Occupation** (**for more than one job report the job with the highest score):**

0 = No job = This category includes those who are not currently in paid work, such as students, retirees and not working, housewife (for women).

1 = Unskilled & manual workers = manual labor requiring minimal specialized skills or training (e.g., street vendors, waste collectors, sentinels/guards, agricultural workers, fishermen, porters, home servants, construction workers, etc.).

2 = Skilled workers = Occupations that require substantial skill or training, such as car drivers, batchers, chose makers, small shops’ sellers, electrician, care repair, carpenter, plumber, trades & small business owners, miners, industrial workers, printing.

3 = Army & police personnel (excluding commanders & officers).

4 = Business & sales personnel = This involves buying and selling items including aspects connected with business such as management, administration, etc.

5 = Office & administrative work = such as office managers, secretaries, administrative assistants, and office clerks.

6 = Professionals = An expert who is mastering a specific type of profession having specialized knowledge in field which he is practicing professionally such as university staff, physicians, schoolteachers, lawyers, accountants, army & police officers.
