# Supplementary material for: Refining and validation of Family Socioeconomic Status Scale (FSESS) for health research in Egypt
Source: BMC Public Health. 2026 Feb 9;26:728. doi: 10.1186/s12889-026-26282-y (PMC12930555; doi:10.1186/s12889-026-26282-y)
Supplement: Supplementary file 3 — Supplementary Material 3. [file 12889_2026_26282_MOESM3_ESM.docx]

**Supplementary table S1.** Convergent validity and construct reliability of the FSESS

| **Latent construct** | **Number of indicators** | **Composite Reliability (CR)** | **Average Variance Extracted (AVE)** |
| --- | --- | --- | --- |
| Education | 2 | 0.86 | 0.76 |
| Occupation (Jobs/Work) | 2 | 0.78 | 0.55 |
| Family income and possessions | 2 | 0.74 | 0.51 |

**Notes:** Composite reliability values exceeded the recommended threshold of 0.70, indicating adequate internal consistency. Average variance extracted (AVE) values were ≥ 0.50 for all constructs, supporting convergent validity.

### **Supplementary table S2. Household possessions checklist and endorsement frequencies**

| **Item** | **Response format** | **Percentage (%)** |
| --- | --- | --- |
| Refrigerator | Yes / No | 97.2 |
| Smartphone | Yes / No | 94.5 |
| Computer (desktop or laptop) | Yes / No | 65.3 |
| Air conditioner | Yes / No | 58.3 |
| Car | Yes / No | 51.2 |
| Washing machine | Yes / No | 50.1 |
| Additional house (other than primary residence) | Yes / No | 39.8 |
| Agricultural land | Yes / No | 36.6 |
| Shop or livestock ownership | Yes / No | 30.6 |
| Non-agricultural land for housing | Yes / No | 30.1 |

**Supplementary table S3.** Confirmatory Factor Analysis (CFA) model fit indices

| **χ²** | **df** | **χ²/df** | **CFI** | **TLI** | **RMSEA** | **RMSEA 90% CI** | **SRMR** | **PNFI** | **PCFI** |
| --- | --- | --- | --- | --- | --- | --- | --- | --- | --- |
| 8,826.15 | 21 | 420.29 | 0.95 | 0.94 | 0.045 | 0.038–0.052 | 0.037 | 0.63 | 0.67 |

**Notes:** Model fit indices indicate acceptable to good fit according to conventional criteria. Parsimony-adjusted indices (PNFI and PCFI) suggest an adequate balance between model fit and model complexity given the large sample size.
